# Understanding MHC Class I Presentation of Viral Antigens by Human Dendritic Cells as a Basis for Rational Design of Therapeutic Vaccines

**DOI:** 10.3389/fimmu.2014.00182

**Published:** 2014-04-23

**Authors:** Nadine van Montfoort, Evelyn van der Aa, Andrea M. Woltman

**Affiliations:** ^1^Department of Gastroenterology and Hepatology, Erasmus MC University Medical Center Rotterdam, Rotterdam, Netherlands

**Keywords:** virus, human dendritic cell, cross-presentation, CTL priming, MHC class I-antigen presentation, viral immunity, immunotherapy, virus–host interaction

## Abstract

Effective viral clearance requires the induction of virus-specific CD8^+^ cytotoxic T lymphocytes (CTL). Since dendritic cells (DC) have a central role in initiating and shaping virus-specific CTL responses, it is important to understand how DC initiate virus-specific CTL responses. Some viruses can directly infect DC, which theoretically allow direct presentation of viral antigens to CTL, but many viruses target other cells than DC and thus the host depends on the cross-presentation of viral antigens by DC to activate virus-specific CTL.

Research in mouse models has highly enhanced our understanding of the mechanisms underlying cross-presentation and the dendritic cells (DC) subsets involved, however, these results cannot be readily translated toward the role of human DC in MHC class I-antigen presentation of human viruses. Here, we summarize the insights gained in the past 20 years on MHC class I presentation of viral antigen by human DC and add to the current debate on the capacities of different human DC subsets herein. Furthermore, possible sources of viral antigens and essential DC characteristics for effective induction of virus-specific CTL are evaluated.

We conclude that cross-presentation is not only an efficient mechanism exploited by DC to initiate immunity to viruses that do not infect DC but also to viruses that do infect DC, because cross-presentation has many conceptual advantages and bypasses direct immune modulatory effects of the virus on its infected target cells.

Since knowledge on the mechanism of viral antigen presentation and the preferred DC subsets is crucial for rational vaccine design, the obtained insights are very instrumental for the development of effective anti-viral immunotherapy.

## Role of Dendritic Cells in the Induction of Anti-Viral Immunity

Immune responses to viral infections are a complex interplay between the virus, target cells, and cells of the immune system. Effective viral clearance requires the induction of virus-specific CD8^+^ cytotoxic T lymphocytes (CTL), which have the capacity to eradicate the virus by direct and indirect mechanisms (1). DC, a low frequent population of white blood cells play a central role in the induction of virus-specific CTL, since they are the most potent antigen presenting cells and unique for their capacity to activate naïve T cells (2). DC are located at strategic positions at sites of pathogen entry, where they continuously sample the environment for invading pathogens. Capturing antigens in combination with encountering danger signals from pathogens induces maturation of DC and their migration to secondary lymphoid organs where they can activate naïve T cells. Activation of naïve CD8^+^ T cells and polarization toward effective CTL requires presentation of MHC class I–peptide complexes (signal 1) together with co-stimulation (signal 2) and the presence of cytokines (signal 3) such as IL-12 (3) and IFNα (4).

Dendritic cells comprise a family of different subsets, diverging in ontogeny, localization, and phenotype. Each DC subset has its own specialized immune functions with regard to the functional interactions with all kind of immune cells, including T cells, B cells, and NK cells, due to differential expression of receptors and intrinsic differences in their ability to produce different cytokines and other membrane-bound and soluble immune modulatory molecules (5). Human DC subsets present in blood, peripheral, and lymphoid tissues can be classified in two main categories: plasmacytoid DC (pDC) and myeloid DC (mDC), which can be further divided into BDCA1^+^ (CD1c^+^) and BDCA3^+^ (CD141^+^) DC (6). pDC are specialized in the production of high amounts of anti-viral type I interferon (IFN; IFNα/β) upon activation (7), whereas BDCA1^+^ DC are known for their high production of IL-12 and their ability to induce T cell responses (5). BDCA3^+^ DC, on the other hand, can produce high levels of type III IFN (IFNλ) (8), which possess direct anti-viral activity, and induce Th-1 responses (9). In the skin, two additional mDC subsets have been characterized, epidermal Langerhans cells (LC) and dermal interstitial DC (intDC) (10). Since DC represent a very rare population in the human body that hampers isolation of sufficient numbers, *in vitro*-generated DC differentiated from monocytes (11) or hematopoietic progenitor cells (12) are frequently used for functional studies on human DC.

The notion that DC compared to other antigen presenting cells stand out in their capacity to induce strong virus-specific CTL goes back more than 20 years, when it was reported that human blood-derived DC exposed to HIV-1 or influenza virus could induce proliferation of autologous CTL (13, 14). At that time, it was not known whether the efficacy of DC reflected specialized antigen presentation pathways or that other factors were responsible for the efficacy of DC in virus-specific CTL cell induction. At least it was noted that only low numbers of DC were sufficient to induce influenza-specific T cells (14).

Now we know that DC, in addition to their broad expression of pattern-recognition receptors (PRR) and excellent T cell stimulatory capacities, harbor unique specialized antigen presentation pathways, that are of major importance for their central role in the induction of virus-specific immunity; DC can efficiently facilitate MHC class I presentation of endogenously synthesized antigens, a process that is active in all nucleated cells, but also facilitate MHC class I presentation of antigen engulfed from exogenous sources, a process called cross-presentation (15). DC are very efficient in capturing exogenous antigen, because they express a diverse repertoire of receptors and exploit various mechanisms to engulf antigens, including endocytosis, phagocytosis, and pinocytosis. The cross-presentation capacity of DC may be crucial for the induction of virus-specific CTL during infections with viruses that do not infect DC.

Seminal mouse studies have demonstrated the importance of cross-presentation for the generation of virus-specific CTL responses (16–18). In addition, mouse studies have provided important insights into the cell-biological mechanisms underlying cross-presentation by DC (19, 20). However, composition of the human DC compartment and susceptibility to viruses differ largely between mice and men. In addition, the mechanism of cross-presentation by human DC is less well-understood. Therefore, research on MHC class I presentation of viral antigens by human DC is of great importance to understand the induction of virus-specific CTL in humans.

The study into antigen presentation of viruses by subsets of human DC *ex vivo* has been facing several technical challenges, which has hampered the understanding of this process for many viruses. However, some recent technical advancements have become available that empowered this research. For example, the possibility to more efficiently isolate human DC subsets from peripheral blood and other organs and the development of a new generation of protocols to generate human DC subsets *in vitro* (21, 22), as was previously shown for BDCA1^+^ monocyte-derived DC (moDC) (11) and CD34^+^ HPC-derived intDC and LC, that resemble mDC found in mucosal tissues including skin (12, 23). These technical advancements have revived the scientific interest in the interactions between viruses and different human DC subsets. Since 2010, a significant body of literature has been published on presentation of viral antigens by different human DC subsets that facilitated this review, which is based for a large part on studies using human DC.

In the present review, the different mechanisms employed by human DC to facilitate MHC class I presentation of viral antigens are discussed. For this purpose, possible sources of viral antigens, essential DC characteristics for optimal MHC class I presentation of viral antigens, and host factors important for virus-specific CTL induction are defined. Furthermore, the roles of the various human DC subsets of human DC in these processes are evaluated. Since knowledge on mechanisms of virus-specific CTL induction by human DC subset is crucial for rational vaccine design, recommendations for development of effective anti-viral immune therapies will be provided based on the insights obtained in this review.

## Sources of Viral Antigen for MHC Class I Presentation by DC

Virus-infected DC can use endogenously synthesized viral proteins as antigens for presentation in MHC class I, whereas non-infected DC need to actively engulf exogenous viral antigens for cross-presentation. Here, we discuss possible sources of viral antigen obtained from different viruses for MHC class I presentation by human DC.

Human moDC are permissive for quite a number of viruses including measles virus (MV), human cytomegalovirus (HCMV), influenza A virus (IAV), human T-cell lymphotropic virus type 1 (HTLV-1), dengue virus (DV), vaccinia virus (VV), respiratory syncytial virus (RSV), herpes simplex virus (HSV), and human metapneumovirus (hMPV) (24–36). Although moDC can take up HIV-1, they are largely refractory to HIV-1 productive infection (37), whereas, productive infection of peripheral blood-derived BDCA1^+^ DC and pDC has been demonstrated (38). In addition to moDC, RSV also infects BDCA1^+^ and BDCA3^+^mDC (39) and IAV infects BDCA1^+^ mDC, but not pDC (40). LC are permissive for MV, but only after maturation (25). Although LC can take up HIV-1, they are not permissive for HIV-1 replication and transmission, but rather prevent it by degradation (41). Permissiveness to infection indicates that these viruses not only enter human DC, they also induce a certain level of protein neo-synthesis in DC that ranges from restricted synthesis of early viral proteins (33) to extensive synthesis of multiple viral proteins and secretion of viral progeny (26). Intracellular synthesis of viral antigens by DC suggests that these infected DC may facilitate direct presentation of viral antigens in MHC class I and activation of virus-specific cytotoxic T cells (CTL). MHC class I presentation of viral antigens has been reported for DC infected with IAV, MV, HTLV-1, and HCMV, albeit sometimes with low efficiency (14, 25, 27, 31, 42).

Nevertheless, it has been demonstrated in several independent studies, involving IAV, HIV-1, and MV, that the efficiency of MHC class I-antigen presentation of replication-incompetent virus was at least comparable to replication-competent virus (25, 40, 43–46). These heat-or UV-treated replication-incompetent viruses have lost the capacity to induce synthesis of viral proteins, but still efficiently enter DC to act as exogenous sources of viral antigen. It was estimated that MHC class I presentation of replication-incompetent IAV by BDCA1^+^mDC was 300 times more efficient than MHC class I presentation of replication-competent IAV (40). These studies clearly show that, at least for the viruses studied, endogenous synthesis of viral antigens is not required for MHC class I presentation and that cross-presentation is an efficient mechanism to facilitate MHC class I presentation of viral antigens.

Thus, cross-presentation is not only an efficient mechanism exploited by DC to initiate immunity to viruses that do not infect DC but also contributes to initiation of anti-viral immunity to viruses that do infect DC. In fact, cross-presentation seems a clever way to bypass direct immune modulatory effects of the virus on its infected target cells. For instance, interference with MHC class I presentation is commonly used by herpes viruses to evade immunity [reviewed by Ref. (47)] and is also exploited by IAV, as was elegantly shown by comparing CMV-specific CTL proliferation by CMV-antigen loaded IAV-infected and uninfected BDCA1^+^ mDC (40). In addition, early during HIV infection, part of the DC compartment is depleted, which may contribute to decreased activation of adaptive immunity (48). Virus-induced cell death is also reported for RSV (34, 39) and VV (33).

**Figure 1:**
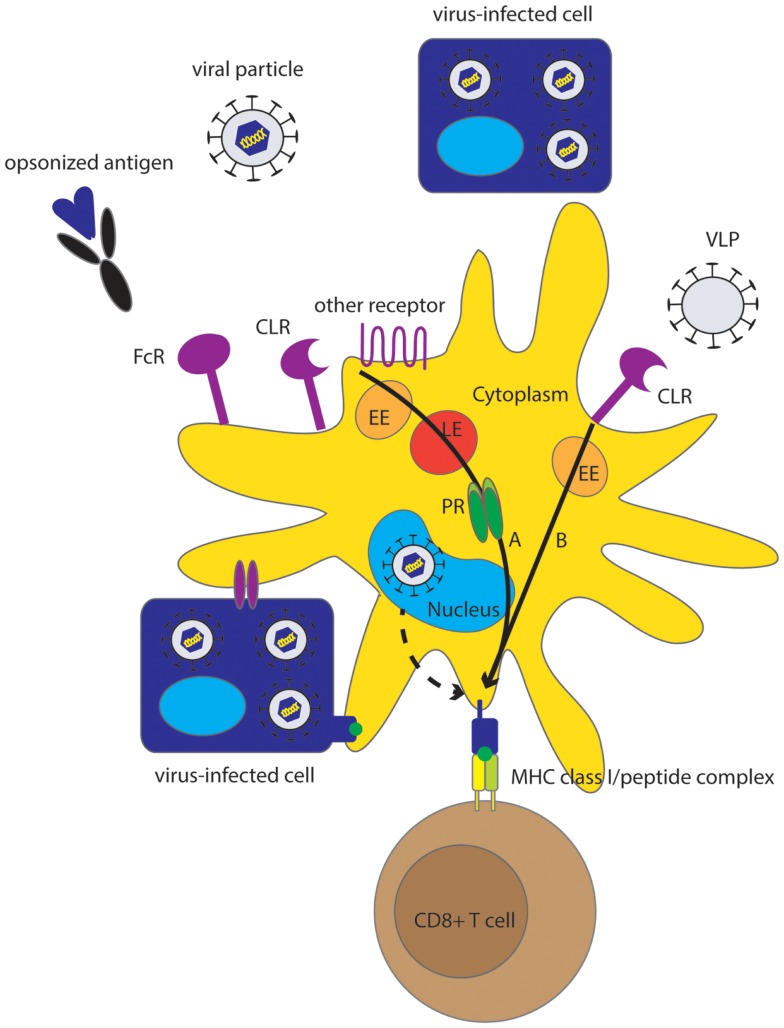
**Overview of different pathways underlying MHC class I presentation of viral antigens by human DC**. Although direct MHC class I class I presentation may contribute to virus-specific CTL induction (dashed arrow), cross-presentation is an effective mechanism for MHC class I presentation of viruses that do not infect DC but also for those viruses that do infect DC. Sources of viral antigen that can be efficiently cross-presented by human DC include viral proteins, (infectious) viral particles, VLP, and virus-infected cells, also referred to as cell-associated Ag. Endocytic receptors including CLR, FcR and other receptors (Table 1) play an important role in the uptake of Ag for cross-presentation. Cross-presentation can be enhanced by opsonization. Two main pathways for cross-presentation have been described that are also relevant for cross-presentation of viruses by human DC and are characterized by differences in the mechanism of protein degradation and differences in kinetics (black arrows). The slower cytosolic pathway, that relies on proteasomal degradation in the cytosol, is important for cross-presentation of viral particles, infected cells, and opsonized viral proteins **(A)**. The relatively fast vacuolar pathway is independent of proteasomal degradation and is important for cross-presentation of VLP **(B)**. Alternatively, DC can obtain viral peptides or MHC class I-peptide complexes by interaction with virus-infected cells. EE, early endosome; LE, late endosome; PR, proteasome.

In addition to replication-incompetent viral particles, other sources of exogenous viral antigens for cross-presentation by human DC include virus-like particles (VLP), viral proteins, and virus-infected cells (Figure 1). VLP morphologically and immunologically resemble infectious viral particles because they contain the natural viral envelop proteins, however, they are not infectious, because they do not contain the viral genome. Although some VLP naturally occur *in vivo*, they are often man-made, being used as safe representatives of viral particles to study virus–host interactions (49) or in the context of vaccine research (50, 51). VLP can be efficient sources of exogenous viral antigen for cross-presentation by DC, as was demonstrated for hepatitis C virus (HCV) VLP (49), human papilloma virus 16 (HPV16) VLP (50), and VLP composed of the coat protein of papaya mosaic virus (PapMV) (51).

Recombinant proteins such as HCV-derived NS3 (52), HIV-1-derived Nef (53), HCMV-derived pp65 (9, 54), and hepatitis B virus (HBV)-derived hepatitis B surface antigen (HBsAg) (55, 56) are sources of exogenous antigens that are often used to study the mechanism of cross-presentation by DC. Nevertheless, the efficiency of cross-presentation of these recombinant proteins is relatively low compared to other sources of viral antigens. Moreover, with the exception of HBsAg, which is secreted by human hepatocytes and can be measured in peripheral blood, most proteins are not naturally occurring as soluble proteins *in vivo* but are only present in/associated with infected cells.

Cell-associated antigen, i.e., antigen associated to or present in infected target cells, represents another important source of viral antigens that can be encountered by DC. Albert and colleagues contributed the first evidence of this by showing that uptake of apoptotic IAV-infected monocytes by moDC leads to efficient activation of influenza-specific CTL (57). After this study, a compelling number of studies have confirmed that virus-infected target cells can be efficient antigen sources for cross-presentation in many infections. For instance, VV-infected monocytes (45, 58), HTLV-1 infected CD4^+^ T cells (31), MV-infected B cell lines (25), HCMV-infected fibroblasts (27, 59), and EBV-transformed B cells (60, 61) are reported as efficient sources of viral antigens for cross-presentation by human DC. The latter study nicely illustrated the high efficiency of this mechanism by demonstrating activation of EBV-specific CTL by DC cross-presenting EBV latency antigens that were expressed at low levels in EBV-transformed B cells (61).

In the above-mentioned studies, apoptotic or necrotic virus-containing cells or cell remnants were used as sources of cell-associated antigens for cross-presentation. Transfer of viral peptides from infected cells to DC could represent an alternative efficient mechanism underlying cross-presentation of cell-associated viral antigens. Two different mechanisms facilitating peptide exchange between cells have been described, including transfer of antigenic peptides via intercellular communication channels, called gap junctions (62), and direct transfer of MHC class I/peptide complexes from infected cells to DC, named cross-dressing (63, 64). The relevance of these pathways in presentation of viral antigens by human DC and induction of virus-specific T-cell immunity should be further evaluated.

In summary, for efficient viral antigen presentation to CD8^+^ T cells, DC can acquire viral antigens from various sources. Although direct presentation of endogenously generated antigen by virus-infected DC has been reported for some viruses, evidence to support an important role for this mechanism in the induction of virus-specific CTL is lacking. In contrast, there is compelling evidence that cross-presentation of exogenously acquired viral antigen is highly efficient and provides an excellent way for the host to bypass evasion mechanisms that several viruses employ to prevent direct MHC class I presentation in infected target cells.

## Endocytic Receptors Involved in Uptake of Viruses by DC

Being intracellular parasites, viruses use the host machinery for internalization, proliferation, and transmission. DC are attractive target cells for viral entry because they express numerous receptors at their cell surface and they migrate through the body, which facilitates viral dissemination. Viruses can enter DC via docking with their viral envelop to endocytic receptors expressed at the cell membrane (43, 44, 46). A commonly described receptor used by viruses to enter DC is DC-specific C-type lectin dendritic cell-specific intercellular adhesion molecule-3-grabbing non-integrin (DC-SIGN/CD209). DC-SIGN is involved in the infection of moDC by DV (32, 65), HCMV (28), HSV (66), MV (67), and IAV (68) and also in DC-mediated transmission of HIV-1 (69) and HTLV-1 (70) to CD4^+^ T cells. DC-SIGN is part of the large family of C-type lectin receptors (CLR), comprising Ca^2+^-dependent receptors that each have unique functions but share the recognition of carbohydrate structures present on micro-organisms (71). Other CLR family members involved in interaction with viruses include Langerin (CD207), involved in the interaction with MV and HIV-1 (25, 41), DC immunoreceptor (DCIR) (72), proposed as an alternative receptor for HIV-1 promoting infection in *cis* and *trans* and macrophage mannose receptor (MMR/CD206), possibly involved in uptake of HBsAg by liver BDCA1^+^ DC (73). Also non-CLR can be involved in the interaction with viruses or VLP. DC-specific heparin sulfate proteoglycan Syndecan-3 cooperates together with DC-SIGN to facilitate infection of DC and transmission to CD4^+^ T cells (74) and is involved in the interaction with HPV VLP (75). Since expression of endocytic receptors varies widely between DC subsets (Table 1), the different subsets will likely have specialized roles in the interaction with different viruses, determined by the combination of receptors expressed on each DC subset.

**Table 1:** **Summary of receptors that are involved in DC–virus interaction on different DC subsets**.

Family	Name	BDCA1^+^ mDC	BDCA3^+^ mDC	pDC	Epidermal LC	Dermal intDC	moDC	Reference
C-type lectin receptors	DEC-205 (CD205)	+	+	+	−	+	+	MacDonald et al. (76), Ebner et al. (77)
	DCIR (CLEC4A)	+	−	+	+	+	+	Bates et al. (78), Lambotin et al. (79), Eklöw et al. (80), Klechevsky et al. (81)
	MMR (CD206)	±	+	−	−	+	+	Chatterjee et al. (82), MacDonald et al. (76), Lambotin et al. (79)
	DC-SIGN (CD209)	−	−	−	−	+	+	Turville et al. (83), MacDonald et al. (76)
	CLEC9A (DNGR1)	−	+	−	−	−	−	Huysamen et al. (84)
	Langerin (CD207)	−	−	−	+	−	−	Turville et al. (83), MacDonald et al. (76)
Toll-like receptors	1	+	+	+	+	+	+	Kadowaki et al. (5), Jongbloed et al. (9), Lambotin et al. (79)
	2	+	+	−	+	+	+	
	3	+	+	−	+	+	+	
	4	+	−	−	−	+	+	
	5	+	−	−	−	+	+	
	6	+	+	+	+	+	+	
	7	−	−	+	+	+	−	
	8	+	+	−	+	+	+	
	9	−	−	+	−	−	−	
	10	+	+	+	−	−	+	
Fcγ receptors	FcγRI (CD64)	+	−	nf	nf	nf	±	Flinsenberg et al. (85)
	FcγRIIA (CD32)	+	+	+	nf	nf	+	Flinsenberg et al. (85), Tel et al. (86)
	FcγRIII (CD16)	−	−	−	nf	nf	−	Flinsenberg et al. (85)
Complement receptors	CR4 (CD11c)	+	+	−	+	+	+	MacDonald et al. (76), Lambotin et al. (79)
	CR3 (CD11b)	±	−	−	±	+	+	Donaghy et al. (87), Lui et al. (88), Poulin et al. (21)
Heparan sulfate proteoglycan	Syndecan-3	nf	nf	nf	nf	nf	+	de Witte et al. (74)
Chemokine receptor	XCR1	−	+	−	−	−	−	Crozat et al. (89), Bachem et al. (90)

*pDC, plasmacytoid DC; LC, Langerhans cell; intDC, interstitial DC; moDC, monocyte-derived DC; nf, information not found*.

Are these CLRs only involved in supporting viruses to enter the host or did they evolve to support activation of the host’s immune system through antigen presentation? Langerin is an important receptor for interaction with pathogens in the skin and has been shown to support antigen presentation in MHC class II, but its role in MHC class I-mediated antigen presentation is under debate (25). Moris et al. showed that blocking of DC-SIGN partly reduced MHC class I presentation of internalized HIV-1 by DC, arguing in favor of a role of DC-SIGN in cross-presentation of HIV-1 (91). In contrast, Sabado et al. showed that blocking of DC-SIGN, DEC-205 (CD205), or MR did not reduce MHC class I presentation of HIV-1 antigens (46) whereas Tjomsland and colleagues showed that blockade of MR even promoted cross-presentation of HIV-1 by DC (92). Thus, the physiological role of DC-SIGN in cross-presentation of HIV-1 is thus far inconclusive, which may be explained by differences in experimental set-up such as the HIV-1 strain used. Antibody-mediated delivery of antigen to the CLRs MR, DEC-205 (82), DCIR (81), DC-SIGN (93), and CLEC9A (94) (Table 1) on human DCs facilitates efficient cross-presentation. These examples show that CLR can facilitate cross-presentation, however, the physiological role of these receptors in cross-presentation of viral antigens is still under debate.

Whereas CLR can directly recognize viral envelop antigens, complement receptors and Fc receptors (FcR) selectively recognize viral antigens that are opsonized with complement and immunoglobulins, respectively. Antigen immune complexes naturally exist and are formed when pre-existing antibodies bind to blood-borne antigens in the circulation, for example, during HCMV re-infection (85). Binding of immune complexes to Fcγ receptor (FcγR) on DC leads to efficient cross-presentation in MHC class I (85). Strikingly, the observation that FcR-dependent uptake of HBsAg can enhance activation of HBV-specific CTL was made years before the concept of cross-presentation by DC was recognized (95), indicating that opsonization of viral antigens may be important for generating virus-specific CTL. Similarly, opsonization of antigen by complement can efficiently enhance cross-presentation, as was recently demonstrated for HIV-1 by targeting HIV-1 particles to CR3 (92). In addition, although not classically referred to as opsonization, binding of high-density lipoprotein (HDL) to HCV VLP supported efficient Scavenger receptor B-mediated uptake and cross-presentation (96). A similar role for extracellular heat-shock proteins (HSP) has been proposed [reviewed by Ref. (97)], mainly based on mouse studies in the field of cancer immunotherapy. However, the role of HSP in cross-presentation of viral antigens by human DC remains to be investigated.

Although these results indicate that several endocytic receptors may be involved in facilitating cross-presentation, their exact role needs to be determined. Especially recognition of viral antigens by opsonins seems to be an effective way of natural antigen targeting to DC for cross-presentation. Increased knowledge on the receptors used by viruses for infection on the one hand and the receptors that facilitate cross-presentation on the other hand may be of great value for therapeutic interventions.

## Mechanisms Underlying Cross-Presentation

One of the intriguing aspects of cross-presentation is that processing of incoming antigen needs to be very efficient to compete with the vast amount of endogenous proteins for MHC class I binding. In addition, cross-presentation requires access of incoming antigen to the MHC class I pathway that is mechanistically separated from the uptake vesicles (98).

Dendritic cells harbor unique pathways to facilitate these logistic and mechanistic challenges underlying cross-presentation. Based on research of numerous groups, two main models have been put together for the mechanisms underlying cross-presentation of exogenous antigens, referred to as the “cytosolic” pathway and the “vacuolar” pathway [reviewed by Ref. (20)]. These pathways are not mutually exclusive and may operate together in one cell (99). The most discriminative aspects between the two pathways are discussed below.

In the cytosolic pathway, antigens are degraded by the proteasome, a large enzyme complex situated in the cytosol that makes this pathway sensitive to inhibitors of proteasomal degradation. Alternatively, in the vacuolar pathway, both antigen degradation and MHC class I presentation occur in the endocytic compartment. Involvement of this pathway can be experimentally addressed by confirming resistance to inhibition of proteasomal degradation and sensitivity to inhibition of lysosomal proteolysis.

Lysosomal proteolysis has a detrimental role in the cytosolic cross-presentation pathway. It was experimentally demonstrated that limiting lysosomal proteolysis by chemically increasing the lysosomal pH favors cross-presentation of viral proteins HCV-derived NS3 and HIV-derived Nef by preventing complete degradation of potential MHC class I binding epitopes (53). Several different adaptations on the endocytic compartment, including a differential lysosomal protease activity, mechanisms to control the lysosomal pH, and antigen storage compartments, together endow DC to facilitate cross-presentation via the cytosolic pathway (100–102). Cross-presentation via the cytosolic pathway further requires export of internalized antigens from the endocytic compartment to the cytosol for proteasomal degradation, which is probably the rate-limiting step in this pathway, at least for protein antigen. Many enveloped viruses can enter the cytoplasm as part of their infection strategy that requires fusion of the viral envelope with the endosomal membrane to release the viral genome into the cytoplasm. This endosomal fusion capacity probably underlies the efficiency of cross-presentation of viral particles, at least for those particles that are able to enter the cytoplasm of DC. The mechanism of cytosolic delivery for other viral antigens and viruses that do not undergo endosomal fusion in human DC is largely unknown. Candidate proteins that may be involved in cytosolic delivery include HSP and p97 and sec61, which belong to the endoplasmic reticulum-associated protein degradation (ERAD) machinery (20), however, the role of these molecules in human DC is poorly studied.

Interestingly, the cytosolic and vacuolar pathway has totally different kinetics, which can be used to determine which pathway is involved (103). Whereas cross-presentation via the vacuolar pathway is fast and can be detected after 20 min (104), cross-presentation via the cytosolic pathway is much slower and formation of MHC class I–peptide complexes via this pathway may take at least 8 h (100), probably because it relies on MHC class I neo-synthesis (20). In contrast, MHC class I loading in the vacuolar pathway occurs in the endocytic compartment and depends on recycling of MHC class I molecules that are constitutively internalized by a highly regulated process (105).

## Viral Road to Cross-Presentation

The cytosolic and the vacuolar pathways were largely established based on model antigens and mouse studies. It is important to assess if these models are applicable to cross-presentation of viral antigens by human DC.

As discussed above, viral particles use receptors expressed on the plasma membrane to enter DC and uptake of viruses often involves endocytosis. After receptor-mediated endocytosis, the cargo is transported through the endocytic compartment, a highly regulated network of vesicles with different characteristics and functions (103). An important function of the endocytic system is to sort internalized receptors and cargo to different locations for either degradation or recycling. Viruses use the endocytic system to exert their fusion capacity, however, at the same time DC use it to obtain viral antigen for cross-presentation. For example, when IAV reaches late endosomes, the low pH enforces conformational change, leading to hemagglutinin-mediated fusion of the endosomal and viral membranes and release of the viral RNA and proteins into the cytoplasm (106). IAV is efficiently cross-presented, at least when its fusogenic activity is intact (43, 107). The fusion dependence was also observed for HIV; cross-presentation of HIV-1 was completely absent when fusion-incompetent HIV-1 mutants were used or fusion was inhibited chemically (44, 46). Cross-presentation of HIV-1 viral particles is sensitive to proteasome inhibitors, but enhanced by inhibition of lysosomal proteolysis (46). Taken together, the above-mentioned work suggests a role for the cytosolic pathway in cross-presentation of fusion-competent viral particles, at least by mDC. Interestingly, cross-presentation of IAV by pDC is not sensitive to proteasome inhibitors, but is sensitive to inhibition of endosomal processing. Together with fast MHC class I presentation, this study suggests a role for the vacuolar pathway for cross-presentation of IAV by pDC.

Evidence from different studies involving IAV-infected monocytes (108), HCMV-infected fibroblasts (27), and EBV-transformed B cells (61) suggests that cross-presentation of cell-associated antigen involves uptake by receptor-mediated phagocytosis and that antigen processing is dependent on the proteasome, but also sensitive to inhibition of lysosomal proteolysis (109). Cross-presentation of Ag–Ig immune complexes also requires both proteasomal and endosomal antigen processing (85). Taken together, these data indicate that although cross-presentation of both cell-associated antigen and Ag–Ig immune complexes require proteasomal degradation, they may need some degree of lysosomal proteolysis to facilitate translocation of antigens from lysosomes to cytoplasm. Since these sources of viral antigen do not have intrinsic fusogenic capacity, they rely on functional specializations of DC to export Ag of the endocytic compartment to the cytosol (103).

Interestingly, several lines of evidence suggest that VLP follow a different pathway for cross-presentation. Cross-presentation of PapMV VLP, HCV VLP, and HBV VLP was not affected by proteasome inhibitors but sensitive to reagents that inhibit lysosomal proteolysis (51, 96, 110). Furthermore, it was shown that cross-presentation of HBV VLP by both mouse DC (110) and human DC (our own unpublished observations) is fast and TAP-independent. Together, these studies suggest that cross-presentation of VLP occurs via the vacuolar pathway.

The differences in cross-presentation pathways between fusion-competent viruses and VLP suggest that different vesicles within the endocytic compartment are involved. Chatterjee et al. showed that antigen targeting via MR or DEC-205 both lead to cross-presentation via different compartments (82). Evidence for a process of sorting comes from an elegant study by Lakadamyali et al., where it was shown that after endocytosis, IAV is sorted into a population of dynamic endosomes that rapidly becomes more acidic, which is necessary for the virus to enter the cytoplasm (111). In contrast, an alternative non-viral ligand, transferrin is sorted into a different population of static endosomes that facilitate recycling of antigen and receptors to the cell surface.

Antigen targeting to DC-SIGN can result in trafficking to different cellular compartments, as was shown for HCV envelop protein and Lewis X uptake via DC-SIGN (112). In addition, antibody-mediated antigen targeting to the neck region of DC-SIGN was dramatically more efficient with regard to cross-presentation of the targeted antigen compared to targeting to the carbohydrate-binding domain, and these differences were related to different endocytic trafficking (93). Taken together, these studies suggest that endocytic sorting is important for the fate of antigens and that sorting occurs at the receptor level. The nature of the sorting signal and the role of endocytic receptors and their adaptor molecules in this process remains to be further elucidated. However, an indication that poly-ubiquitination may be involved in sorting and antigen translocation comes from a mouse study involving the MMR (113).

We conclude that both the cytosolic and the vacuolar pathways are applicable to cross-presentation of viral antigen by human DC, depending on the type of viral antigen that is encountered by DC (Figure 1). The studies discussed above suggest that VLP preferentially traffic via the vacuolar pathway for cross-presentation, whereas protein antigen, fusion-competent viral particles, cell-associated antigen, and Ig-opsonized antigen preferentially traffic via the cytosolic pathway for cross-presentation, except in pDC that may preferentially facilitate the vacuolar pathway. Since the above-mentioned studies together suggest that antigen is sorted into pathways with different efficiency of cross-presentation at the receptor level, it is of high importance to gain more knowledge on the receptors used for internalization of viral antigens and their exact role in the sorting of Ag to different pathways in order to fully understand the cross-presentation of viral antigens. Currently, besides VLP, no other viral antigens were found that utilize the vacuolar cross-presentation pathway in human mDC, thus the physiological role of this pathway remains to be further understood. However, since this pathway is highly efficient, as was demonstrated in pDC (114), further understanding of the mechanisms underlying the vacuolar pathway may be of interest for therapeutic purposes.

## DC Maturation as a Critical Factor for CTL Induction

Antigen presentation in MHC class I can lead to CTL priming or tolerance, depending on the context in which DC encounter the antigen (15). Sensing of danger signals by PRR on DC (Table 1) induce DC maturation, a differentiation process initiated after innate immune recognition that regulates key functions involved in CTL induction, including migration, antigen presentation, co-stimulation, and production of cytokines. Co-stimulation lowers the threshold for antigen recognition by the T-cell receptor and is important for proliferation, survival, effector function, and memory formation of T cells. Changes in antigen presentation after DC maturation include upregulation of MHC class I molecules (42), enhanced proteasomal activity (115), and reduced lysosomal antigen degradation (116) due to lower expression of lysosomal proteases (107). It is well-accepted that matured human DC have an enhanced capacity to activate virus-specific CTL (25, 42, 56, 60, 117, 118). Importantly, however, the experimental stimuli used for induction of DC maturation are often not representative for the type of danger signals that are encountered by DC during viral infection *in vivo*.

Which danger signals can be naturally encountered by PRR on DC during viral infection? Viruses can display danger signals of various nature including viral nucleic acids, replication intermediates, carbohydrate structures, and proteins on the envelop, that can be sensed by PRR on DC (Table 1). IAV and RSV, both ssRNA viruses, induce maturation of different human DC subsets including moDC, BDCA1^+^ mDC, and pDC (34, 39, 42, 119, 120). Also VLP have been shown to induce DC maturation (49, 50, 75), which is not dependent on TLR but may be mediated by a recently identified innate recognition mechanism (121). In addition to virus-derived danger signals, virus-induced danger signals produced by the host in response to viral infection can induce DC maturation. Examples of such virus-induced host-derived maturation signals include cytokines such as IFNα/β and TNFα secreted by virus-infected cells (122) and damage-associated molecular patterns (DAMP) released by damaged or dying cells (123). During interaction of DC with cell-associated Ag, DC can encounter both virus-derived danger signals and host-derived maturation signals (27, 124, 125) or host cell-derived DAMP, such as TLR4 ligand high-mobility group box 1 (HMGB1) (126) or CLEC9A ligand F-actin (127).

The induction of DC maturation by virus-derived and virus-induced stimuli suggests that these factors also enhance CTL priming, however, direct experimental evidence on the contribution of virus-induced DC maturation on CTL induction by human DC is limited. IAV-infection of DC is associated with strong DC maturation and efficient antigen-specific CTL proliferation (42, 117). Similarly, TLR agonist poly I:C that mimics viral double-stranded RNA (dsRNA) is a strong inducer of DC maturation and effectively enhances cross-presentation of recombinant viral antigen by several subsets of human DC (9, 56, 128, 129). Also TLR7/8 agonists have been shown to enhance DC-induced CTL expansion and effector function *in vitro* (81). In contrast, cross-presentation of cell-associated antigen was inhibited when polyI:C or IAV were present in the captured dead cells, suggesting that virus-derived danger signals may also have a detrimental effect on cross-presentation, which may be specific for cross-presentation of cell-associated antigen (130). IFNα, a widely studied representative of virus-induced signals, can exert multiple effects on human DC that promote CTL cross-priming [reviewed by Ref. (4)]. For example, moDC differentiated in the presence of IFNα, so called IFNα-DC, have superior cross-presentation capacity compared to classical moDC (52, 131). In conclusion, although it is widely accepted that virus-derived and virus-induced stimulatory signals are required for effective cross-priming of virus-specific CTL, it has been difficult to experimentally address this hypothesis in the currently used *in vitro* models. Challenges include the low precursor frequency of naïve virus-specific CD8^+^ T cells and dissection of the separate contributions of DC maturation and antigen presentation to CTL induction.

Interference with DC maturation and thereby subverting the development of effective CTL induction is an important mechanism of immune evasion used by many viruses. Examples of viruses that interfere with DC maturation are MV (132), VV, via the production of cytokine receptor homologs (33), HSV, via destabilization of host mRNA (35, 133) and HCMV, which prevents upregulation of co-stimulatory molecules and production of cytokines (134) and induces TGFβ production by its target cells (124). Furthermore, DC isolated from patients with chronic HIV, HBV, and HCV infections showed functional impairments in the capacity to produce IL-12 or induce T-cell activation, which may be a direct effect of the virus on DC and thereby the cause of the failing adaptive immune response, but could also be the consequence of the chronic infection (135, 136).

The connection between innate immune recognition of viruses by human DC and the induction of virus-specific CTL is an important subject for further study. In addition, the PRR and pathways underlying recognition of viruses by DC and the mechanisms by which viruses circumvent these pathways needs to be further explored. Novel molecular techniques such as the ability to knock down PRR in human DC will empower this research, which is important for the development of therapeutic interventions.

## DC Subsets Involved in Cross-Presentation of Viral Antigen

Before 2010, the large majority of studies on cross-presentation of viral antigen by human DC were performed with *in vitro*-generated moDC, however, more recently a number of groups have succeeded in obtaining sufficient numbers of DC from blood or other organs to assess the ability and mechanism of cross-presentation of viral antigens by different human DC subsets.

BDCA3^+^ DC were initially recognized as a subset with superior cross-presentation capacity compared to other human DC subsets (9, 21, 89, 137). Comparison of transcriptional profiles revealed that BDCA3^+^ DC represent the human equivalent of murine CD8α^+^ and CD103^+^ DC (56, 138), which have a superior intrinsic cross-presentation capacity compared to other DC subsets (139). In parallel, selective expression of CLEC9A (84), a receptor that senses dead cells (140) and facilitates cross-presentation by mouse (141) and human DC (94), suggested that human BDCA3^+^ DC would excell in cross-presentation of dead cell material. Superior capacity to cross-present cell-associated antigen by BDCA3^+^ DC was demonstrated by several independent studies (9, 21, 89, 102, 137), however, not observed in all studies (118). Although BDCA3^+^ DC are highly capable of cross-presenting cell-associated antigen, cross-presentation of cell-associated antigen has also been demonstrated for BDCA1^+^ DC (102), pDC (89, 118), and moDC (31, 57). Also for other types of antigen, cross-presentation is not restricted to the BDCA3^+^ DC subset. Cross-presentation of protein antigen was shown for peripheral blood and tissue-derived BDCA1^+^ DC (9, 128), BDCA-2^+^ pDC (102, 128), and BDCA3^+^ DC (9, 56, 102, 128, 137), as well as for *in vitro*-generated CD34^+^-derived DC (102) and moDC, as discussed above. Although BDCA3^+^ DC are highly capable of cross-presenting cell-associated antigen, cross-presentation of cell-associated antigen has also been demonstrated for BDCA1^+^ DC (102), pDC (89, 118), and moDC (54).

Both BDCA3^+^ and BDCA1^+^ DC share the specialized machinery that is associated with efficient cross-presentation capacity, i.e., high phagosomal pH, production of ROS within endocytic compartments, and efficient transfer of exogenous antigens into the cytosol (102). Both subsets have a similar efficiency of endogenous MHC class I presentation after transfection, a similar efficiency of cross-presentation of heat-inactivated IAV that can egress to the cytosol at low pH and a similar efficiency of cross-presentation of antigen that is selectively delivered to early endosomes (107). Nevertheless, BDCA3^+^ DC were superior compared to BDCA1^+^ DC at cross-presentation of antigen that was artificially targeted to lysosomes by using antigen conjugated to DEC-205 targeting antibodies (107). This suggests that although both DC subsets can efficiently cross-present Ag delivered to early endosomes, BDCA3^+^ DC may exhibit a specialized machinery to transfer Ag from late endosomes and lysosomes to the cytosol. This DC characteristic might explain the superior capacity to cross-present IgG-opsonized antigen targeted to FcγR that could not be attributed to superior FcγR expression and/or antigen uptake in these cells (85).

Plasmacytoid DC contribute to anti-viral immune responses by producing large amounts of IFNα/β, however, their role as professional antigen presenting cell in the initiation of virus-specific T-cell responses was initially questioned based on controversial results in mice (86). Direct comparison of intrinsic characteristics that can influence cross-presenting capacity, such as phagosomal pH and ROS production, between pDC and BDCA1^+^ and BDCA3^+^ mDC was hampered due to inconclusive data for pDC (102). However, pDC express a broad repertoire of antigen-uptake receptors on their cell surface such as FcR and CLR BDCA-2, DEC-205, DCIR that can facilitate the uptake and cross-presentation of viral antigens (116) (Table 1). In addition, pDC can efficiently transfer exogenous Ag into the cytosol suggesting that they may be capable of cross-presenting antigen via the cytosolic pathway (102). Numerous functional studies showed that human pDC can cross-present recombinant protein antigens, long peptide antigens, IAV-derived antigens, and cell-associated antigens (88, 118, 119, 142). In addition, it was also demonstrated that pDC can efficiently cross-present viral antigen via the vacuolar pathway, which may be facilitated by MHC class I storage in recycling endosomes (114). Taken together, we conclude that human pDC can efficiently facilitate cross-presentation of a wide range of viral antigens. Direct comparison of cross-presentation efficiency between human pDC and mDC was thus far inconclusive, with one study showing a higher efficiency of cross-presentation by pDC (114), another study showing superior MHC class I-restricted IAV presentation by BDCA1^+^ mDC (40) and three studies concluding that pDC and BDCA1^+^ or BDCA3^+^ mDC have similar cross-presentation efficiencies (118, 119, 142).

Although blood DC required DC maturation for efficient cross-presentation, skin or lymph node DC can cross-present under steady state conditions, which might be due to a more mature/activated status of these tissue DC compared to circulating DC (56, 102, 143). In addition to BDCA1^+^ and BDCA3^+^ DC, skin contains Langerin^+^ LC and dermal intDC, often referred to as CD14^+^ DC. Comparison of CD14^+^ DC to other skin DC subsets indicated that this subset showed the least cross-presenting capacity among skin subsets (10, 56), which may be related to the finding that these cells express immunoglobulin-like transcript receptors that antagonize CTL development (144). Cross-presentation capacity of LC cells is under debate and may vary upon the source of LC and type of antigen used in experiments. Cross-presentation of recombinant protein antigen by *in vitro*-generated LC has been demonstrated in several independent studies (10, 102, 145), however, cross-presentation of replication-incompetent MV and MV-infected cells by skin-derived LC was absent (25). Sine LC are potentially interesting vaccine target cells, because of their presence at mucosal sites such as skin and higher respiratory tract (25), further studies on the cross-presentation capacity of primary LC are required.

We conclude that essential mechanisms of cross-presentation are present among most human DC subsets, with the exception of CD14^+^ DC. Superiority of cross-presentation among DC subsets can be attributed to the repertoire of uptake receptors and adaptations in the endocytic compartment and may vary depending on the type of antigen.

## Technical Limitations and Novel Approaches

Although several technical advancements have potentiated the study of MHC class I-antigen presentation by human DC, several important questions remain to be addressed.

One of the current technical challenges is to measure antigen presentation at the level of DC. The purest read-out would be to measure MHC class I-antigen complexes at the surface of DC (signal 1 only), however, tools are lacking (20). The best current available method to quantify MHC class I-antigen presentation is a read-out involving activation or *in vitro* induction of virus-specific T cells. However, it should be taken into account that activation of virus-specific T cells results from a combination of TCR ligation by MHC class I–peptide complexes (signal 1) and other stimuli provided by DC such as cytokines and co-stimulation (signal 2 and 3).

The study of induction of human CD8^+^ T cells by DC is also hampered by the extreme low frequency of naïve virus-specific T cells in peripheral blood. As discussed above, MHC class I presentation by human DC has been most frequently studied for IAV, HIV-1, and CMV. For these viruses, it has been possible to obtain sufficient numbers of “memory” T cells from peripheral blood and use T-cell expansion and IFNγ production as read-outs for antigen presentation in an autologous setting (13, 14, 54). Virus-specific T-cell clones to other viruses can be obtained by several rounds of antigen-specific expansion *in vitro*. However, performance of such *in vitro*-generated clones in cross-presentation studies is complicated due to their limited life span and the allogenic bias present in experiments because DC and T cells are not from the same donor. A novel promising approach for the study of cross-presentation of viruses by human DC is the use of T-cell receptor transfer to generate autologous virus-specific T cells (146, 147). Such T cells are evaluated in the context of immunotherapy of patients but may also be exploited as tools to monitor antigen presentation by DC.

## Recommendations and Considerations for Development of Therapeutic Vaccine Strategies

Chronic viral infections such as HIV, HBV, and HCV are a big health burden and affect 100 millions of patients worldwide. Viral persistence is associated with a failure of the patient’s immune response to eradicate the virus (136). In addition to chronic persistent infections, reactivation of latent infections including HCMV, EBV, and HPV is a major threat for immune compromised patients. In addition, a high proportion of these chronic and latent infections including HIV, HBV, HCV, EBV, HPV, and HTLV is related to the development of malignancies later in life (148). Immunotherapy represents an attractive therapeutic intervention to combat such infections and prevent virus-related malignancies by using the body’s own defense mechanisms. To accomplish this, immunotherapy is directed to improve virus-specific immunity and eradicate the virus but also generate protective memory responses to prevent re-infections. Moreover, immunotherapy should overcome T-cell exhaustion and anergy, often observed in patients with chronic infections (148).

Insights into the mechanisms underlying effective priming of virus-specific CTL by human DC are instrumental for the development of effective virus-specific immunotherapy. We identified cross-presentation as a crucial mechanism for the induction of virus-specific CTL and embrace the concept to utilize the effective cross-presentation mechanisms naturally present in DC for immunotherapy. In line with this concept, antibody-mediated antigen targeting to endocytic receptors is an emerging approach employed by numerous groups to target antigen to DC for cross-presentation. Endocytic receptors that efficiently facilitate cross-presentation by human DC include FcγRIIA, CLEC9A, DEC-205, and DCIR (81, 85, 94, 116, 149). An advantage of antigen targeting to specific receptors is the possibility to select receptors that are uniquely expressed by distinct subsets of DC (Table 1), such as proposed for XCR1 (150) or CLEC9A (94). Selective targeting to DC prevents antigen consumption by irrelevant cells, which may lead to reduced availability of antigen to DC and improper T-cell activation.

As discussed previously, DC maturation is crucial for virus-specific CTL induction. Although the endocytic receptors are very potent in internalizing antigen, their role in promoting DC maturation is less clear. Therefore, the combination of antigen targeting with adjuvants is an important field of study. FcγR have been shown to facilitate both efficient antigen uptake and DC maturation, however, it was recently shown that FcγR-dependent DC maturation in human DC is less strong than was previously observed in mice DC (85, 151). Other interesting approaches that combine antigen targeting to DC and DC maturation in one cargo include TLR-ligand–peptide conjugates (152) and nanoparticles that contain both antigen and adjuvant (116).

Since DC comprise a heterogeneous family of subsets that differ in location, frequency, receptor expression, and functional specializations, it is important to design a therapeutic vaccine with the desired DC subset in mind. Based on accumulated evidence from *in vitro* studies on antigen presentation by human DC subsets, we conclude that most human DC subsets have the basic capacity to cross-present, as long as the antigen is efficiently targeted to an endocytic compartment that favors cross-presentation. Nevertheless, DC subsets do have unique functional characteristics, such as type of cytokine production, which can have high impact on the type of immune response induced. Moreover, DC subsets express different PRR (Table 1) and only adjuvants for a selected number of TLRs are currently available at clinical grade.

In addition to antigen targeting to DC *in vivo*, recruiting of DC precursors may represent an attractive immunotherapeutic approach, as was recently proposed for monocytes, which can contain a natural reservoir of HBsAg that can be presented in MHC class I upon differentiation of these monocytes to moDC (153).

## Concluding Remarks and Future Perspectives

Based on two decades of research into MHC class I-restricted presentation of viral antigen by human DC, we conclude that cross-presentation of viral antigens is a highly efficient mechanism for defense against viruses. Furthermore, cross-presentation of viral antigens seems not only pivotal for defense against viruses that do not infect DC, but also for those that infect DC, as demonstrated by *in vitro* studies using replication-incompetent IAV, HIV-1, and MV. Since these viruses represent a selection of all viruses that can productively infect human DC, the contribution of direct presentation by human DC infected with other viruses cannot be completely ruled out. Nevertheless, as discussed in this review, cross-presentation has many conceptual advances compared to direct presentation by infected DC.

So far, knowledge on the presentation of viral antigens by human DC is mainly derived from *in vitro* studies. Whether these studies faithfully represent the *in vivo* situation is of course difficult to predict. Several caveats from these *in vitro* studies include the use of *in vitro*-generated DC, which may behave differently than their *in vivo* counterparts, the use of laboratory adapted virus strains, and pseudo-typed viruses, which may have tropisms that may not represent the *in vivo* situation, and the use of recombinant viral proteins and TLR ligands that are not fully representative for antigens or danger signals that can be encountered *in vivo*. Nevertheless, taking these limitations into account, together these studies have given us an important understanding of the mechanisms underlying MHC class I presentation of viral antigens by human DC. This knowledge is an important basis for the rational design of therapeutic vaccines for chronic viral infections.

Interesting venues for further research include identification of DC receptors involved in viral infection and initiation of immune response, elucidation of the molecular signals underlying sorting of viral antigen to endocytic compartments that favor cross-presentation and the role of virus-derived danger signals and virus-induced maturation stimuli in cross-presentation and CTL priming.

A more detailed knowledge of these key factors in virus–host interaction will further empower the design of novel therapeutics for infectious diseases.

## Conflict of Interest Statement

The authors declare that the research was conducted in the absence of any commercial or financial relationships that could be construed as a potential conflict of interest.

## Abbreviations

DC, dendritic cell; CTL, cytotoxic T lymphocyte; mDC, myeloid dendritic cell; pDC, plasmacytoid dendritic cell; moDC, monocyte-derived dendritic cell; LC, Langerhans cell; PRR, pattern-recognition receptor; VLP, virus-like particle; CLR, C-type lectin receptor; FcR, Fc receptor; TLR, Toll-like receptor.
